# Plasmid-Assisted Horizontal Transfer of a Large *lsa*(E)-Carrying Genomic Island in Enterococcus faecalis

**DOI:** 10.1128/spectrum.00154-22

**Published:** 2022-07-06

**Authors:** Xinxin Shan, Xin-Sheng Li, Stefan Schwarz, Yuxia Chen, Chunyan Xu, Xiang-Dang Du

**Affiliations:** a College of Veterinary Medicine, Zhengzhou, People’s Republic of China; b Institute of Microbiology and Epizootics, Centre for Infection Medicine, Department of Veterinary Medicine, Freie Universität Berlin, Berlin, Germany; c Veterinary Centre of Resistance Research (TZR), Freie Universität Berlin, Berlin, Germany; USDA-ARS

**Keywords:** *Enterococcus faecalis*, genomic island, conjugative plasmids, Tn*916*, horizontal transfer

## Abstract

The horizontal transfer of genomic islands is essential for the adaptation and evolution of Enterococcus faecalis. In this study, three porcine E. faecalis strains, each harboring a large *lsa*(E)-carrying genomic island, were identified. When using the E. faecalis OG1RF as the recipient, the horizontal transfer of the *lsa*(E)-carrying genomic island occurred only from E. faecalis E512, which also harbored a pheromone-responsive conjugative plasmid, but not from the other two E. faecalis strains, E533 and E509, which lacked such a plasmid. Subsequently, through plasmid curing of E. faecalis E512 and plasmid introduction into E. faecalis E533, the pheromone-responsive conjugative plasmid was identified to be indispensable for the horizontal transfer of the *lsa*(E)-carrying genomic island and a subsequent homologous recombination between the chromosomal DNA of the donor and the recipient. In addition, the presence of a chromosomally-located conjugative transposon, Tn*916*, in E. faecalis E509 could not mediate the horizontal transfer of the *lsa*(E)-carrying genomic island, although Tn*916* itself could transfer by conjugation. Thus, these data highlight the role of the pheromone-responsive conjugative plasmid in the transfer of the *lsa*(E)-carrying genomic island in E. faecalis, thereby establishing the dual role of pheromone-responsive conjugative plasmids in contributing to the dissemination of both plasmid-borne resistance genes and chromosomally-located genomic islands.

**IMPORTANCE** In this study, it was shown that a pheromone-responsive conjugative plasmid played an indispensable role in the horizontal transfer of a *lsa*(E)-carrying genomic island. This finding indicates a dual role of the pheromone-responsive conjugative plasmid in disseminating both plasmid-borne resistance genes and chromosomally-located genomic islands. The role of the pheromone-responsive conjugative plasmid in disseminating chromosomal genomic islands is suggested to be essential in the genomic evolution of E. faecalis, which has become one of the leading nosocomial pathogens worldwide.

## INTRODUCTION

Antimicrobial-resistant bacteria are a major cause of health care-associated infections around the world, and resistance has emerged in infections in the wider community (1). Antimicrobial-resistant pathogens, particularly multiresistant ones, are an increasing, major health care problem worldwide. Both Gram-negative and Gram-positive bacteria face the evolutionary challenge of antimicrobial chemotherapy, which is often overcome via the acquisition of preexisting resistance determinants from the respective bacterial gene pool (1). Enterococci are the third leading cause of hospital-associated infections and have gained increased importance due to their fast adaptation to the clinical environment via the acquisition of antimicrobial resistance and pathogenicity traits (2). Enterococcus faecalis is a common commensal but is also a relevant nosocomial pathogen and a leading cause of hospital-acquired infections (3). Horizontal gene transfer has contributed to the evolution of enterococci, transforming them into leading causes of hospital-acquired infections (4, 5). The vancomycin-resistant strain V583 was the first sequenced E. faecalis genome, and over a quarter of it consists of mobile elements and/or exogenously acquired DNA, including probable integrated phage regions, insertion elements (IS), multiple conjugative and composite transposons, a putative pathogenicity island, and integrated plasmid genes (6, 7). Comparison of the genomes of 38 E. faecalis strains revealed no distinct differences which would distinguish between clinical and nonclinical E. faecalis genomes (8).

The enterococcal ABC-F gene *lsa*(E) that confers combined resistance to lincosamides, pleuromutilins, and streptogramin A antibiotics was first reported in 2013 in methicillin-susceptible and methicillin-resistant Staphylococcus aureus (9). The *lsa*(E) gene was located in a multiresistance gene cluster, along with the genes *aadE*, *spw*, and *lnu*(B) (9–11). To date, multiple variants of *lsa*(E)-carrying multiresistance gene clusters have been found among streptococci, enterococci, staphylococci, and other Gram-positive bacteria (9–15). In most cases, the *lsa*(E)-carrying gene cluster is located on conjugative or mobilizable plasmids, and its transfer mainly depends on the transfer of the corresponding plasmids. However, if the *lsa*(E)-carrying gene cluster is located in the chromosomal DNA, its mode of transfer remains unknown.

In the current study, we investigated the mechanism of transfer of *lsa*(E)-carrying genomic islands in three E. faecalis strains: E512, E533, and E509. Among them, E. faecalis E512 harbored a pheromone-responsive conjugative plasmid, E. faecalis E509 carried the conjugative transposon Tn*916*, and E. faecalis E533 lacked both plasmids and Tn*916*. This study was initiated to address the following three questions: (i) Does the transfer of chromosomal *lsa*(E)-carrying genomic islands in E. faecalis occur? (ii) What is the transfer mechanism of *lsa*(E)-carrying genomic islands in E. faecalis? (iii) What are the essential factors for the successful transfer of *lsa*(E)-carrying genomic islands in E. faecalis?

## RESULTS

### The *lsa*(E) gene is part of large chromosomal genomic islands in three E. faecalis isolates.

**FIG 1:**
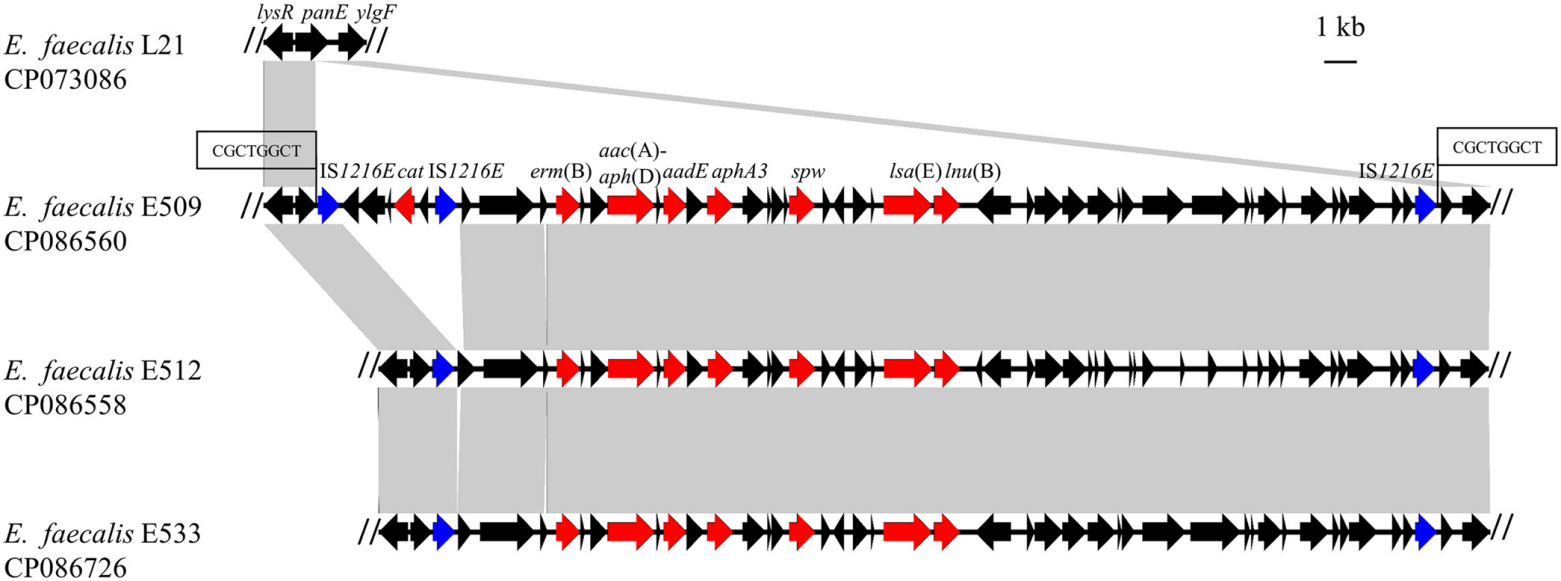
The structure of the *lsa*(E)-carrying genomic islands in E. faecalis isolates E509, E512, and E533 as well as their comparison with E. faecalis L21. Antimicrobial resistance genes are shown in red, IS*1216E* in blue, and other genes in black.

**FIG 2:**
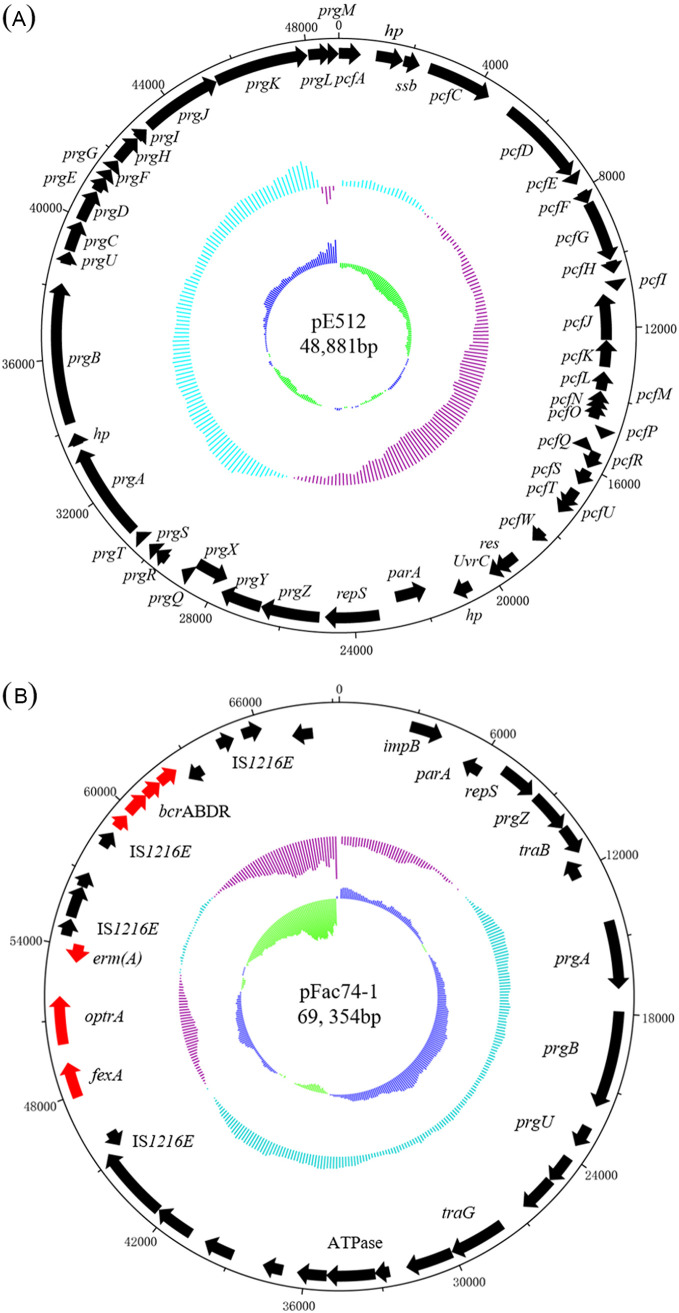
The structure of the plasmids, pE512 from E. faecalis isolate E512 (A) and pFac74-1 from E. faecalis isolate Fac74 (B). The circles display (from the outside to inside): (i) the size scale in bp; (ii) the positions of predicted coding sequences, transcribed in the clockwise orientation; (iii) the positions of predicted coding sequences, transcribed in the counterclockwise orientation; (iv) the GC content plotted against 50%, with blue indicating >50% and purple indicating <50%; and (v) GC skew [(G-C)/(G+C)] in a 1,000 bp window. Antimicrobial resistance genes are shown in red, and other genes in black.

In this study, the three *lsa*(E)-positive E. faecalis strains, E509, E512, and E533, were used for analyses (Tables 1 and 2). Whole-genome sequencing (WGS) and corresponding analyses revealed that E. faecalis E512 carried a *lsa*(E)-positive genomic island and a pheromone-responsive conjugative plasmid, which were 30,536 bp and 48,881 bp in length, respectively (Fig. 1 and 2A). The pheromone-responsive conjugative plasmid is a *rep9*–type plasmid (pCF10 prototype, known to be the best studied pheromone-responsive conjugative plasmids), designated pE512. This plasmid harbors the essential pheromone-responsive genes (*prg*), such as *prgA*, *prgB*, and *prgZ*, as well as the type IV secretion system (T4SS) genes, such as *prgC*, *prgF*, *prgK*, *prgG*, and *prgT* (16). The *lsa*(E)-positive genomic island had inserted via IS*1216E* into the chromosomal *panE* gene, encoding a ketopantoate reductase of E. faecalis E512, and generating 8 bp direct target duplications (5′-CGCTGGCT-3′) at the integration site. Other resistance genes, including *erm*(B) (combined resistance to macrolides, lincosamides, and streptogramin B), *aac*(A)-*aph*(D) (resistance to gentamicin and tobramycin), *aad*(E) (resistance to streptomycin), *aphA3* (resistance to kanamycin and neomycin), *spw* (resistance to spectinomycin), and *lnu*(B) (resistance to lincosamides) were also identified in the *lsa*(E)-carrying genomic island (Fig. 1).

**TABLE 1:** The strains used in this study

Strains		Characteristics			
	Descriptions	Resistance phenotype	Conjugative plasmid	Tn*916*	*Isa*(E)
E512	Wild-type strain (ST69)	ERY, LIN, GEN	pE512	−	+
E512-TC1	Transconjugant from E512×OG1RF	ERY, LIN, GEN, RIF, FUS	pE512	−	+
E512-TC2	Transconjugant from E512×OG1RF	ERY, LIN, GEN, RIF, FUS	pE512	−	+
E512-PC	E512 derivative cured of the conjugative plasmid pE512	ERY, LIN, GEN		−	+
Fac74	Wild-type strain (ST1243)	FFC, ERY, LIN, GEN	pFac74-1	−	−
Fac74-TC	Transconjugant from Fac74×JH2-2	FFC, LIN, GEN	pFac74-1	−	−
E533	Wild-type strain (ST69)	ERY, LIN, GEN		−	+
E533-PI	E533 derivative with introduction of conjugative plasmid pFac74-1	FFC, ERY, LIN, GEN	pFac74-1	−	+
E533-PI-TC	Transconjugant from E533-PI×OG1RF	FFC, ERY, LIN, GEN, RIF, FUS	pFac74-1	−	+
E509	Wild-type strain (ST16)	ERY, LIN, GEN, TET		+	+
E509-TC	Transconjugant (Tn*916*^+^) from E509×OG1RF	TET, RIF, FUS		+	−
OG1RF	Recipient strain	RIF, FUS		−	−
JH2-2	Recipient strain	RIF, FUS		−	−

**TABLE 2:** Antimicrobial susceptibility testing (AST) results of the wild-type strains, transconjugants, and recipient strains used in this study

	MICs (mg/L)^*a*^										
Strains	RIF R ≥ 4	FUS /	GEN HLAR ≥500	ERY R ≥ 8	CHL R ≥ 32	TET R ≥ 16	FFC /	CLI /	TML /	VAN R ≥ 32	BAC /
E512	2	4	>512	>512	128	128	4	512	512	2	64
E512-TC1	>512	>512	>512	>512	4	<1	2	512	512	4	32
E512-TC2	>512	>512	>512	>512	4	<1	2	512	512	4	32
E512-PC	2	4	>512	>512	128	128	4	512	512	2	32
Fac74	<1	2	>512	>512	128	128	64	512	256	2	>512
Fac74-TC	512	512	128	<1	32	<1	64	32	256	2	>512
E533	2	2	>512	>512	128	128	2	512	512	2	64
E533-PI	2	2	>512	>512	>512	128	64	512	512	2	>512
E533-PI-TC	>512	>512	>512	>512	4	<1	2	512	256	2	>512
E509	<1	2	>512	>512	128	64	4	512	256	2	64
E509-TC	>512	>512	128	<1	4	64	2	32	256	2	32
OG1RF	>512	>512	128	<1	4	<1	4	32	256	2	32

a RIF, rifampicin; FUS, fusidic acid; GEN, gentamicin; ERY, erythromycin; CHL, chloramphenicol; TET, tetracycline; FFC, florfenicol; CLI, clindamycin; TML, tiamulin; VAN, vancomycin; BAC, bacitracin; R, resistance; HLAR, high-level resistance to aminoglycosides.

E. faecalis E509 carried a similar *lsa*(E)-positive genomic island, with a length of 34,087 bp, which harbored a similar set of additional resistance genes as the corresponding genomic island of E. faecalis E512, but, in addition, harbored a *cat* gene, encoding chloramphenicol resistance (Fig. 1). In addition, E. faecalis E509 carried a chromosomally-located conjugative Tn*916* transposon carrying the tetracycline resistance gene, *tet*(M).

E. faecalis E533 harbored a similar *lsa*(E)-carrying genomic island as E. faecalis E512 with a length of 30,578 bp (Fig. 1) but no additional plasmids or transposons. These three *lsa*(E)-positive, naturally occurring E. faecalis strains provided the opportunity to explore the mechanism and factors underlying the transfer of the *lsa*(E)-positive genomic island in E. faecalis under different conditions, including: (i) the presence of the pheromone-responsive conjugative plasmid (E512), (ii) the presence of Tn*916* (E509), and (iii) a strain lacking both the pheromone-responsive conjugative plasmid and Tn*916* (E533).

### The *lsa*(E) gene is transferable only in one of the three *lsa*(E)-positive E. faecalis strains.

Conjugation experiments were conducted to determine the transferability of the *lsa*(E) gene in the three *lsa*(E)-positive E. faecalis strains. Surprisingly, the *lsa*(E) gene was only transferable from E. faecalis strain E512, which harbored the pheromone-responsive conjugative plasmid. Two transconjugants, E512-TC1 and E512-TC2, were selected for further analysis.

### Chromosome-chromosome homologous recombination results in the transfer of the *lsa*(E)-carrying genomic island in E. faecalis E512.

**FIG 3:**
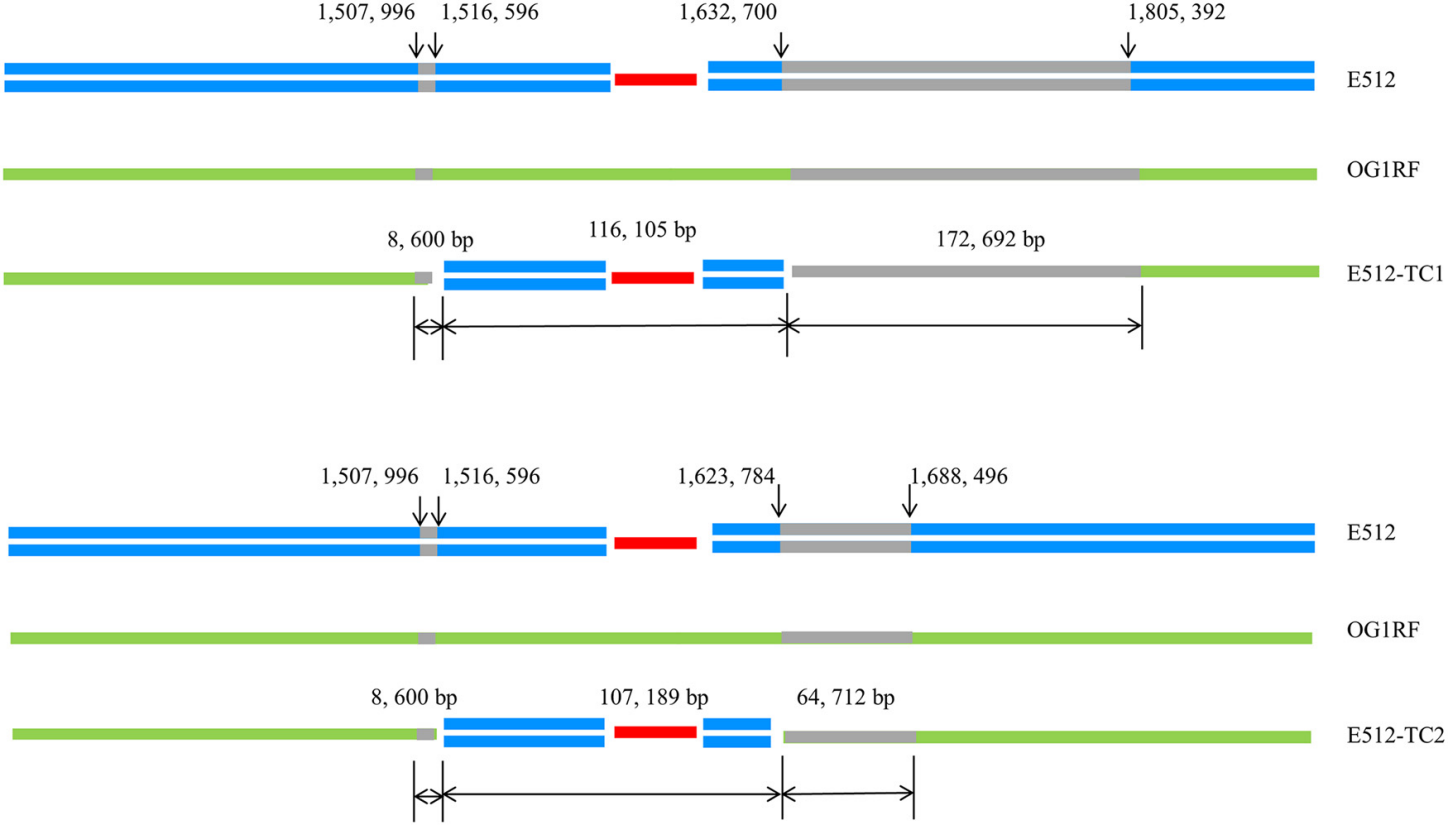
Simplified schematic diagram for the transfer of a large chromosomal segment, including a *lsa*(E)-carrying genomic island in two transconjugants: E512-TC1 and E512-TC2. Transferred chromosomal segments, including a 30,536 bp *lsa*(E)-carrying genomic island (shown in red color), were framed up, which were 116,105 bp in transconjugant E512-TC1 and 107,189 bp in transconjugant E512-TC2. The putative homologous regions that served for the integration of the chromosomal regions of E. faecalis E512 into the recipient E. faecalis OG1RF are shown in gray color. The sizes of those regions were the same on the left-hand side (8,600 bp) but varied on the right-hand side in the two transconjugants, with 172,692 bp in E512-TC1 and 64,712 bp in E512-TC2.

WGS of the transconjugants E512-TC1 and E512-TC2 was performed, and characteristics of the transconjugants are shown in Table 1, Table 2, and Fig. 3. Sequence analysis indicated that transconjugants E512-TC1 and E512-TC2 were generated by a homologous recombination event between the chromosomal DNA of the donor E. faecalis strain E512 and the recipient E. faecalis strain OG1RF, leading to the transfer of the *lsa*(E)-carrying genomic island from the donor into the recipient. We used single nucleotide variant (SNV) analysis to investigate the transferred chromosomal regions using a previously described method for the transfer of the large chromosomal regions carrying the *vanB* and *pbp5* genes between clinical Enterococcus faecium strains (16). In brief, the SNVs were obtained by comparing the chromosomal sequences of the donor E512, recipient OG1RF, and transconjugants E512-TC1 and E512-TC2. The transferred chromosomal regions were located between the last SNV on the left of the 30,536 bp *lsa*(E)-carrying genomic island in E512 and the first SNV on the right of it. These transferred chromosomal regions, including the *lsa*(E)-carrying genomic island, varied in size in the transconjugants E512-TC1 and E512-TC2, which were 116,105 bp and 107,189 bp, respectively (Fig. 3), resembling similar genomic changes to those associated with the previously described transfer of large chromosomal regions in E. faecium (16). The putative homologous regions that served for the integration of the aforementioned chromosomal regions of E. faecalis E512 into the recipient E. faecalis OG1RF were shown in gray color in Fig. 3. The sizes of those regions were the same in the left-hand side (8,600 bp) but varied in the right in the two transconjugants, being 172,692 bp in E512-TC1 and 64,712 bp in E512-TC2 (Fig. 3). Furthermore, the conjugative plasmid in E. faecalis strain E512 was detected in both transconjugants, E512-TC1 and E512-TC2, revealing its potential participation in the transmission process.

### The conjugative plasmid was indispensable in the transfer of the *lsa*(E)-carrying genomic island in E. faecalis, as evidenced by plasmid curing and plasmid introduction.

As the transfer of the *lsa*(E)-carrying genomic island was only observed in E. faecalis E512 that harbored the pheromone-responsive conjugative plasmid pE512, we hypothesized that this plasmid might represent an essential factor in the transfer of the *lsa*(E)-carrying genomic island. Thus, plasmid curing and plasmid introduction experiments were performed to verify this assumption.

**FIG 4:**
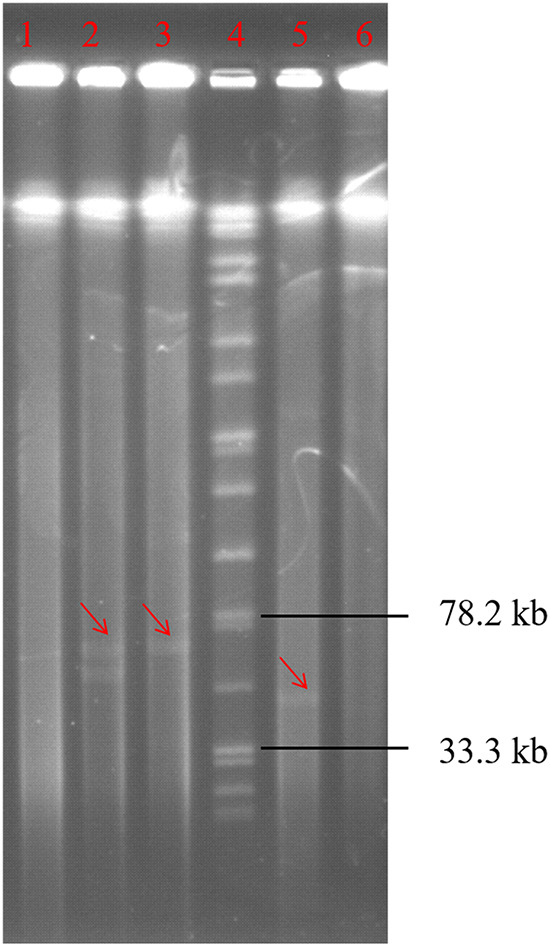
S1-PFGE analysis for the plasmid profile of E. faecalis strains used in plasmid curing and introduction assay. The plasmids’ bands were pointed by red arrows, and the explanations of them are noted following their strain names. Lane 1, E. faecalis E533 (no plasmid); Lane 2 E. faecalis Fac74-TC (pheromone-responsive conjugative plasmid pFac74-1); Lane 3, E. faecalis E533-TC (pheromone-responsive conjugative plasmid pFac74-1); Lane 4, marker; Lane 5, E. faecalis E512 (pheromone-responsive conjugative plasmid pE512); Lane 6, E512-PC (no plasmid).

To determine the effects of the pheromone-responsive conjugative plasmid pE512 on genomic island transfer, a derivative of E. faecalis E512, deficient in the conjugative plasmid and designated E512-PC, was obtained by plasmid curing using novobiocin. In a second strategy, the pheromone-responsive conjugative plasmid pFac74-1 (Fig. 2B) was introduced into the plasmid-free E. faecalis E533 and designated E533-PI (Table 1 and 2). This approach was necessary, as pE512 did not carry any resistance genes that could be used as selection markers. In contrast, pFac74-1 carried the florfenicol/oxazolidinone resistance gene *optrA*, the florfenicol resistance gene *fexA*, the macrolide-lincosamide-streptogramin B resistance gene *erm*(A), and the bacitracin resistance operon *bcr*ABDR. Although there are differences between plasmids pFac74-1 and pE512 in the T4SS genes, pFac74-1 is also a pheromone-responsive conjugative plasmid harboring the *prgA*, *prgB*, and *prgZ* genes and can be transferred by conjugation (authors’ own unpublished observation). The map of the conjugative plasmid pFac74-1 is shown in Fig. 2B. To verify either the plasmid curing or the successful conjugation, S1-PFGE for strains E512-PC and E533-PI were performed. The results showed that both the plasmid curing and the plasmid introduction experiments were successful (Fig. 4), meaning that E512-PC no longer harbored the plasmid pE512 and that the pheromone-responsive conjugative plasmid pFac74-1 was successfully introduced into E533-PI.

To determine the transferability of the *lsa*(E)-carrying genomic island, E. faecalis E512-PC and E533-PI were used as donors for genomic island transfer into the recipient strain, E. faecalis strain OG1RF. Unsurprisingly, only the *lsa*(E)-carrying genomic island from E533-PI was transferred, and the corresponding transconjugant was designated E533-PI-TC (Tables 1 and 2). These data suggested that the *lsa*(E)-carrying genomic island can be transferred only in the presence of a pheromone-responsive conjugative plasmid, such as pFac74-1 or pE512. We therefore concluded that the pheromone-responsive conjugative plasmid was indispensable in the transmission process of the *lsa*(E)-carrying genomic island.

### Conjugative transposon Tn*916* did not contribute to the transfer of the *lsa*(E)-carrying genomic island in E. faecalis.

As E. faecalis strain E509 harbored the conjugative transposon Tn*916*, conjugation experiments were also performed, using E. faecalis E509 as the donor and E. faecalis OG1RF as the recipient, to determine whether the presence of Tn*916* could also contribute to the transfer of the *lsa*(E)-carrying genomic island in E. faecalis. However, multiple conjugation attempts indicated that the presence of Tn*916* did not facilitate the transfer of the *lsa*(E)-carrying genomic island, although Tn*916* itself could transfer between the donor and the recipient (transconjugant designated E509-TC) (Tables 1 and 2). Therefore, these results indicated that Tn*916* in E. faecalis strain E509 did not aid in the transmission of the *lsa*(E)-carrying genomic island.

## DISCUSSION

Pheromone-responsive conjugative plasmids represent a unique group of self-transferable, narrow host-range plasmids, mainly described in E. faecalis, and contribute to the genetic exchanges that allow antimicrobial resistance genes to spread within bacterial populations through their transfer systems (17). Prior studies showed that a conjugative plasmid was involved in the transfer of large chromosomal regions and facilitated the acquisition of β-lactam and vancomycin resistance via homologous recombination in E. faecium (16). Pheromone-responsive plasmids had also been reported to promote chromosomal diversification in *E. faecalis*, and mobilize all chromosomally encoded traits queried, including pathogenicity island, capsule, vancomycin and tetracycline resistances (2). In this study, we present evidence that a pheromone-responsive conjugative plasmid was both necessary and sufficient for the transmission of the *lsa*(E)-carrying genomic island in E. faecalis.

Earlier work from our laboratory had reported that *lsa*(E) was present in enterococci of both human and swine origins, revealing that horizontal transfer might play an important role in the dissemination of the *lsa*(E)-carrying multiresistance gene cluster derived from the plasmid pXD4 (11). In this work, we provide evidence that pheromone-responsive conjugative plasmids play an indispensable role in the transmission of chromosomal *lsa*(E)-carrying genomic islands. Previous studies reported that individual transposons had been identified as harboring *vanA*- or *vanB*-carrying elements (18, 19), and the transfer of these determinants between E. faecium strains had been associated with the movement of large segments of chromosomal DNA (20–22). However, while we observed the transfer of Tn*916* itself, this transfer was not accompanied by the simultaneous transfer of the *lsa*(E)-carrying genomic island in our study. In addition, we showed that the integration of the *lsa*(E)-carrying genomic island into the chromosomal DNA of the recipient occurred via homologous recombination between flanking sequences present in the donor and recipient genomes.

To date, there is no categorical evolutionary distinction between clinical, commensal, and animal strains of E. faecalis (23). This suggests that animal-associated E. faecalis, especially multidrug-resistant strains belonging to sequence type ST16, may retain a zoonotic potential that is mainly linked to the lack of host specificity. Our study highlights the importance of further research on the role of pheromone-responsive conjugative plasmids in the transfer of chromosomal DNA segments between clinical and nonclinical strains of E. faecalis and on the acquisition and transfer of multidrug resistance determinants, such as those encoded within the *lsa*(E)-carrying genomic island. One limitation of this study is the use of only one “engineered” laboratory strain (OG1RF) as the recipient in conjugation assays. It is possible that the results shown in this study may change if other strains were used as recipients.

## MATERIALS AND METHODS

### Bacterial strains.

E. faecalis strains E509, E512, and E533 were isolated from rectal swab samples of pigs from a farm in Henan Province, China in 2015. E. faecalis Fac74 was isolated from a rectal swab sample of a pig from a different farm in Henan Province, China in 2017. The isolation method was as described previously (11). E. faecalis Fac74-TC was a transconjugant derived from matings between E. faecalis Fac74 (donor) and E. faecalis JH2-2 (recipient). The detailed information for all strains used in this study is listed in Table 1.

### AST.

AST for rifampicin, fusidic acid, gentamicin, clindamycin, erythromycin, chloramphenicol, tetracycline, and florfenicol were performed using broth dilution methods, according to the recommendations of the Clinical and Laboratory Standards Institute (CLSI). Results were interpreted according to CLSI document M100 (24). Staphylococcus aureus ATCC 29213 and E. faecalis ATCC 29212 were used as quality control strains.

### Plasmid curing.

Conjugative plasmid-deficient derivatives of E. faecalis strain E512 were obtained by plasmid curing using novobiocin (25). E. faecalis E512 harboring the conjugative plasmid pE512 was inoculated into BHI broth (~1 × 10^5^ CFU/mL) with increasing concentrations (0 to 10 mg/L) of novobiocin (Sigma) and incubated at 37°C for 72 h. The culture that grew at the highest novobiocin concentration was serially diluted and plated onto BHA plates to obtain individual colonies. Randomly selected colonies were replica plated onto BHA plates and screened for the pE512-associated gene, *pcfG*, encoding the relaxase/mobilization nuclease domain-containing protein by PCR (Table S1). Plasmid profiles of the E. faecalis strain E512 and its derivatives were prepared and analyzed by agarose gel electrophoresis.

### Plasmid introduction.

In order to introduce the conjugative plasmid into E. faecalis E533, the transconjugant Fac74-TC, containing the conjugative plasmid pFac74-1, stored in our laboratory was used as the donor in mating experiments (11) with the E. faecalis recipient strain E533. Transconjugants were screened on BHA agar plates supplemented with erythromycin (20 mg/L) and florfenicol (16 mg/L). Plasmid profiles of E. faecalis E533 derivatives (after the introduction of the conjugative plasmid) were prepared and analyzed via agarose gel electrophoresis. Moreover, the transformants were investigated by PCR for the presence of the *optrA* and *impB* (encodes ImpB/MucB/SamB family protein) genes, using the primers shown in Table S1, and confirmed by multilocus sequence typing (MLST), following harmonized protocols (https://pubmlst.org/).

### Conjugation assay.

In order to determine the transferability of the *lsa*(E)-carrying genomic islands, conjugation experiments were performed using the methods described previously (11). Three E. faecalis strains (E509, E512, and E533) served as donors in mating experiments with the rifampicin-resistant recipient strain, E. faecalis OG1RF. Transconjugants were screened on BHA agar plates supplemented with erythromycin (20 mg/L) and rifampin (32 mg/L). The transconjugants carrying *lsa*(E) genomic islands were confirmed by PCR, using the primers listed in Table S1 and AST as described above (11).

To investigate whether the transfer of the *lsa*(E)-carrying genomic island could occur in E. faecalis, E. faecalis derivatives E512-PC (cured of the conjugative plasmid) and E533-PI (with the introduced conjugative plasmid) were used as donors with E. faecalis OG1RF as the recipient strain in mating experiments. Transconjugants were screened on BHA agar plates supplemented with erythromycin (20 mg/L) and rifampin (32 mg/L). The resulting transconjugant was designated E533-PI-TC.

To further investigate whether Tn*916* could aid in the transfer of the *lsa*(E)-carrying genomic islands, conjugation experiments were performed by employing previously described methods (11). The E. faecalis wild-type strain E509 was used as the donor in mating experiments with the recipient strain, E. faecalis OG1RF. Transconjugants that were positive for Tn*916* were screened on BHA agar plates supplemented with tetracycline (10 mg/L) and rifampin (32 mg/L). At the same time, transconjugants that were positive for *lsa*(E)-carrying genomic islands were screened on BHA agar plates supplemented with tetracycline (10 mg/L), erythromycin (20 mg/L), and rifampin (32 mg/L).

### S1-PFGE.

In order to identify whether the conjugative plasmid in E. faecalis E512-PC was cured and whether the conjugative plasmid in E. faecalis E533-PI was introduced successfully, E. faecalis strains E512, E512-PC, E533, and E533-PI were digested with S1 nuclease (TaKaRa, Osaka, Japan), followed by pulsed-field gel electrophoresis (PFGE) with the CHEF Mapper XA system (Bio-Rad, CA) (26). The Salmonella enterica subsp. *enterica* serovar Braenderup H9812 standard strain, digested with XbaI, was used as the molecular size marker.

### Genomic DNA extractions and high-throughput sequencing.

The genomic DNA of E. faecalis strains E509, E512, E533, and Fac74, as well as the two transconjugants (E512-TC1 and E512-TC2) was extracted using the Wizard Genomic DNA purification kit (Promega, Beijing, China), following the manufacturer’s instructions. Whole-genome sequencing of these strains was performed via a combination of the Illumina MiSeq 2500 and PacBio RS platforms (Shanghai Personal Biotechnology Co., Ltd., China).

### Bioinformatics analysis.

The sequences from Illumina MiSeq were assembled using Newbler version 2.8 (454 Life Sciences, Branford, CT, USA), and PacBio sequencing reads were assembled with HGAP4 and CANU (Version 1.6) and corrected by Illumina Miseq with pilon (Version 1.22). The predictions of ORFs and their annotations were obtained using Glimmer 3.0. Artemis and Easyfig were used to generate genetic comparison figures (27).

### Data availability.

The genome sequences of E. faecalis strains E509, E512, E533, E512-TC1, and E512-TC2 have been deposited in GenBank under accession numbers CP086560, CP086558, CP086726, CP086564, and CP086566, respectively. Moreover, the sequences of the two plasmids pE512 and pFac74-1 have been deposited in GenBank under accession numbers CP086559 and CP086593, respectively.
