# Supplementary material for: Plasmid-Assisted Horizontal Transfer of a Large lsa(E)-Carrying Genomic Island in Enterococcus faecalis
Source: Microbiol Spectr. 2022 Jul 6;10(4):e00154-22. doi: 10.1128/spectrum.00154-22 (PMC9430800; doi:10.1128/spectrum.00154-22)
Supplement: Supplemental file 1 — Supplemental material. Download spectrum.00154-22-s0001.pdf, PDF file, 0.3 MB [file spectrum.00154-22-s0001.pdf]

Supplementary materials

Table S1. PCR primers used in this study

Category and gene	Primer designation	Sequence (5'-3')	Product size(bp)	Reference or source
lsa(E)	*lsa*(E)-fw	TGTAAAACGGCTTCCTGATG	496	1
	lsa(E)-rv	TGTCAAATGGTGAGCAAACG		
pcfG	*pcfG*-fw	ATTACAAGCAGCAATTCAAC	638	This study
	pcfG-rv	AATTATATCTAGTGGGGTC		
optrA	*optrA*-fw	GCACCAGACCAATACGATACAA	794	2
	optrA-rv	TCCTTCTTAACCTTCTCCTTCTCA		
impB	*impB*-fw	TTGATTATTCCAAAGAACCCC	836	This study
	impB-rv	AATTTGATCCTTTC GAGCAT		

REFERENCE

1. Li XS, Dong WC, Wang XM, Hu GZ, Wang YB, Cai BY, Wu CM, Wang Y, Du XD. 2014. Presence and genetic environment of pleuromutilin-lincosamide-streptogramin A resistance gene *lsa*(E) in enterococci of human and swine origin. J Antimicrob Chemother 69:1424-6.
2. Yang M, Li XS, Li D, Shang Y, Yu R, Schwarz S, Huang Z, Du XD. 2020. Two novel *lsa*(E)-carrying mobile genetic elements in *Streptococcus suis*. J Antimicrob Chemother 75:2689-91.
